# Recent developments in receptor tyrosine kinases targeted anticancer therapy

**DOI:** 10.14202/vetworld.2016.80-90

**Published:** 2016-01-29

**Authors:** Samir H. Raval, Ratn D. Singh, Dilip V. Joshi, Hitesh B. Patel, Shailesh K. Mody

**Affiliations:** 1 Department of Veterinary Pathology, College of Veterinary Science and Animal Husbandry, Sardarkrushinagar Dantiwada Agricultural University, Sardarkrushinagar, Banaskantha - 385 506, Gujarat, India; 2 Department of Pharmacology and Toxicology, College of Veterinary Science and Animal Husbandry, Sardarkrushinagar Dantiwada Agricultural University, Sardarkrushinagar, Banaskantha - 385 506, Gujarat, India

**Keywords:** cancer, monoclonal antibodies, small molecule drugs, receptor tyrosine kinases, targeted therapy

## Abstract

Novel concepts and understanding of receptors lead to discoveries and optimization of many small molecules and antibodies as anti-cancerous drugs. Receptor tyrosine kinases (RTKs) are such a promising class of receptors under the investigation in past three decades. RTKs are one of the essential mediators of cell signaling mechanism for various cellular processes. Transformations such as overexpression, dysregulation, or mutations of RTKs may result into malignancy, and thus are an important target for anticancer therapy. Numerous subfamilies of RTKs, such as epidermal growth factor receptor, vascular endothelial growth factor receptor, fibroblast growth factor receptors, insulin-like growth factor receptor, and hepatocyte growth factor receptor, have been being investigated in recent years as target for anticancer therapy. The present review focuses several small molecules drugs as well as monoclonal antibodies targeting aforesaid subfamilies either approved or under investigation to treat the various cancers.

## Introduction

In multicellular organisms, intercellular and intracellular communication is essential for normal healthy life. Cells should be capable of processing signalment after receiving it from autocrine, paracrine, endocrine, or juxtacrine sources as a response to any change in their surrounding environment. Triggering of cellular response requires transmission of these signals across the plasma membrane to reach the target inside the cells. The cells have evolved a variety of signaling cascade for the transmission of information for important biological processes. Among this cascade, receptor tyrosine kinases (RTKs) are one of the essential mediators of the signaling mechanisms and plays a crucial role in various cellular processes like differentiation, growth and apoptosis in response to different stimuli [1,2]. Recent advances in molecular biology revealed the vital role of RTKs in the pathophysiology of cancer [3,4]. Normally, activities of RTKs are well-regulated in healthy cells, but sometimes their functions are transformed causing malignancy. These transformations are resulting due to over-expression or mutation of their gene and stimulation of autocrine or paracrine activity [5-7]. A constitutive oncogenic activation in neoplastic cells can be inhibited by selective tyrosine kinase inhibitors and thus considered as a promising therapeutics for novel genome-based targeted therapy [5].

In this Review, we discuss oncogenic activation of RTKs followed by several molecules drugs as well as monoclonal antibodies targeting subfamilies of RTKs either approved or under investigation to treat the various cancers.

### Tyrosine Kinases

“Tyrosine kinases” are the protein superfamily having a transmembrane RTKs and non-RTKs as two main groups present in the cell membrane and cytoplasm, respectively [2,3,6,8,9]. Since discovery of the first RTK about three decades ago, many members of this transmembrane proteins family of cell surface receptors regulates vital cellular processes like proliferation and differentiation, cell migration, cell survival and metabolism, and cell-cycle control. Until date, 58 RTKs have been identified, which are divided into 20 subfamilies [2,10]. The RTKs have three distinct domains *viz*. extracellular domain for specific ligand, a signal pass transmembrane hydrophobic helix, and cytoplasmic tyrosine kinase domain [2,11-13].

The RTKs have both cell surfaces transmembrane receptors, as well as cytoplasmic domain having kinase-like enzyme activity [11]. When signaling molecules bind to RTKs, they cause neighboring RTKs to associate with each other, forming cross-linked dimers. Cross-linking activates the tyrosine kinase activity in these RTKs through cross-phosphorylation [11,14]. One of the most common intracellular signaling pathways triggered by RTKs is known as the mitogen-activated protein kinase cascade. This cascade initiates with Ras (monomeric GTPase signaling protein) activation which is normally bound to GDP in its inactive state and becomes activated when bound to GTP. GTP-bound Ras further activates the first serine-threonine kinase (Raf), which in turn activates Mek and then finally Erk is activated by Mek. Several downstream proteins, which include other protein kinases as well as gene regulatory proteins in the nucleus, are phosphorylated by Erk. These results are changes in gene expression, and thus protein activity which cause complex changes in cell behavior [15]. This pathway is used by nerve growth factor, platelet-derived growth factor (PDGF), and other similar factors.

Since RTKs have pleiotropic expressions, they should be carefully regulated. Overexpression of RTKs may has adverse consequences such as neoplasm, developmental abnormalities, fibrosis, and other diseases [16]. Activated RTKs are downregulated by protein-tyrosine phosphatases (PTPs) and/or endocytic mechanisms. PTPs inactivate RTKs by dephosphorylation of the cytoplasmic domain of RTKs [17,18]. Endocytic mechanisms involve removal of the receptors from the membrane, after which they are either recycled or degraded [19-21]. One of the best studied examples for such endocytic mechanism is RTKs trafficking, which involves the regulation of the epidermal growth factor receptor (EGFR) [19].

### Oncogenic Activation of RTKs

Normally, the level of cellular tyrosine kinase phosphorylation is controlled without error, but there are several mechanisms which lead to oncogenic activation of RTKs, which include enhanced signal generation, oncogenic mutations of RTKs, gene amplifications of RTKs, and oncogene fusions involving RTKs. Important examples are discussed as under within the scope of the review.

### Enhanced signal generation

Oncogenic activation of RTKs may occur due to overexpression of their respective ligands and/or adaptor proteins, autocrine-paracrine loop, and co-occurring mutations altering the signaling pathways instigated by RTKs [22-24]. Colony-stimulating factor 1 is secreted by tumor cells, which in response increases macrophages activity within the tumor microenvironment. These events lead to increased production of EGF and thus, more EGF ligand are available for receptor activation. The gene expression of hypoxia-inducing factor 1 is enhanced in hypoxic tumor regions, by tumor-associated macrophages which then regulates vascular endothelial growth factor (VEGF) and causes formation of new blood vessels in the hypoxic area [22]. In cases of melanoma, RTKs such as VEGF receptor (VEGFR), fibroblast growth factor receptors (FGFR), hepatocyte growth factor receptor (HGFR), Insulin-like growth factor-1 receptor (IGF1R), and macrophage-stimulating protein receptor are activated through autocrine signaling [23]. Overexpression of RTKs like ERBB4 is observed in breast tumors. ERBB4 is activated by enhanced activity of sheddase (ADAM17) enzyme. This mechanism involves shedding or cleavage of extracellular domain of ERBB4 and results in tumorigenic ERBB4 activity without the presence of any ligand [24].

### Oncogenic mutations of RTKs

Mutations in the extracellular domain of the RTK gene ERBB2 were found to be of highly oncogenic nature in pulmonary neoplasm. These mutants can be activated by two mechanisms, i.e., either by increased phosphorylation of C-terminal tail or by intermolecular disulfide bond mediated covalent dimerization [25]. In oncogenic activation of ERBB2, NeuT receptors were found to be the result of a Val664Glu mutation in its transmembrane domain. Another mutation at the same residue (Val664Gln) was also found to lead to full oncogenic activation of the receptor [4]. Tandem kinase duplication in the tyrosine kinase domain is another example of oncogenic mutations which has been rationally described in glioblastomas (GBMs). These mutants have active configurations and oncogenic ability [26].

### Gene amplifications of RTKs

A high level intratumoral heterogeneity is characteristics of GBM. This heterogeneity extends to the pattern of RTKs expression and their amplification, for example, PDGF receptor α (PDGFR-α), EGFR, and HGFR [27]. EGFR amplification in breast carcinomas [28] and lung adenocarcinoma [29] are also example of neoplasam involving such RTKs gene amplification.

### Oncogene fusions involving RTKs

Genomic rearrangements cause the formation of oncogenic fusion tyrosine kinases (FTKs) which is composed of activated N-terminal dimerization domain. This activation of domain is induced by the fusion partner protein which is fused to the kinase domain of a receptor or non-RTK (non-RTKs). FTKs are cytoplasmic or occasionally nuclear proteins that do not have extracellular domains, and they have no any response to ligand [30]. Tyrosine kinase fusion genes are reported to be present in 5-7% of hematological, mesenchymal, and epithelial tumors [31].

## RTKs as Targeted Therapy for Cancer

Chemotherapy, surgery and radiation alone or in combinations are mainly used methods for cancer therapy since a long time. The chemotherapeutic drugs target rapidly dividing cells including neoplastic cells and certain normal tissues. The main drawback of these drugs and/or radiation method is the significant toxic effect produced by them. Although classical cytotoxic chemotherapy, alone or in combination with other procedures, remains the treatment of choice for many malignancies, targeted therapies are gaining popularity now as a component of various cancer therapy including breast, colorectal, lung, and pancreatic cancers, as well as lymphoma, leukemia, and multiple myeloma [32].

This targeted therapies are expected to be a more effective and less harmful than systemic chemotherapy because their aim is to block specific pathways related to carcinogenesis and tumor growth by inducing apoptosis of cancer cells, blocking specific enzymes, and growth factor receptors involved in cancer cell proliferation, or modifying the function of proteins that regulate gene expression and other cellular functions, instead of interfering with development of rapidly growing cells [33]. Various distinct targeted therapies have been approved for use in cancer treatment which include, signal transduction inhibitors, gene expression modulator, apoptosis inducer, angiogenesis inhibitor, hormone therapies, immunotherapies, and toxin delivery molecules.

Small molecule drugs and monoclonal antibodies (mABs) are the two main approaches which target the RTKs for cancer therapy. Small molecules are usually organic compounds having low-molecular weight, i.e., <800 dalton, whereas mABs are large molecules. These “small molecules” are able to penetrate the cell membrane and are specifically designed to act on targets found inside the cell or to interfere with signaling pathways [33]. The generic names for the most of small molecule drugs are suffixed with “ib” (gefitinib, lapatinib, etc.) which indicates its inhibitory properties, whereas mABs drugs are suffixed with “mab” (panitumumab, trastuzumab, etc.). They are designated humanized antibodies, which bind to cancer cell-specific antigens.

## Small-molecule Drugs

Afatinib, erlotinib, gefitinib, and lapatinib are synthetic anilinoquinazoline compounds and are example of the small molecule drugs [34-37]. Gefitinib and erlotinib are antitumor drugs, reported to act at the cytosolic ATP binding domain, and thus inhibits autophosphorylation, however, their mechanism of action is not completely unfolded [38,39]. Lapatinib is a reversible RTK inhibitor having activity against ERBB1 as well as ERBB2 [3]. Lapatinib binds the inactive form of EGFR and differs from other EGFR tyrosine kinase inhibitors, such as erlotinib or gefitinib, as later drugs bind EGFR in its active conformation only. This is the reason for a slower dissociation rate of lapatinib when compared to that of erlotinib or gefitinib, resulting into a greater duration of effect at the target site [36].

## mABs

Antibody-based cancer therapy has gained good momentum past in two decades and are now included into one of the successful and important strategies for treating patients suffering with hematological malignancies and solid tumors. Cetuximab, panitumumab, and trastuzumab are naked antibody drug example that targets RTK of the cell membrane. The mechanisms of tumor cell killing by antibodies based drugs may vary *viz*., (i) Direct action of the antibody (through receptor blockade or agonist activity, induction of apoptosis, or delivery of a drug or cytotoxic agent), (ii) immune-mediated cell killing mechanisms which includes complement-dependent cytotoxicity, (iii) antibody-dependent cellular cytotoxicity and regulation of T-cell function, and (iv) specific effects of an antibody on tumor vasculature and stroma [40]. The initial production of mAB was based on immunization of mice with the target antigen. Thus, mAB produced with such technique were comprised mouse protein entirely and were highly antigenic in nature causing hypersensitivity reactions in humans. To overcome these limitations, modern mAB are produced to have a maximum proportion of human components and minimum proportion of murine components, for example, chimeric, humanized and human antibodies contain 65%, 95% and 100% human components, respectively [41]. Suffix of the antibody-based drugs indicates the type of antibody used, e.g., -momab (murine), -ximab (chimeric), -zumab (humanized), and -mumab (human) [32].

## Targeting the Subfamilies of RTKs

Numerous carcinogenic genes are recognized in recent years and are characterized with the help of studies on molecular pathogenesis and complete sequencing of the human genome. About 20% of human genes code products are known to participate in cell signaling pathways [6]. Tyrosine kinases are such a product playing crucial role in coding for enzymatic activities, and they are known as RTKs when act as transmembrane receptors [42]. Important signaling pathways responsible for basic functions of cells *viz*. growth, proliferation, migration, synthesis and apoptosis are stimulated by tyrosine kinases [6]. Activated RTKs can cause increased growth and proliferation of tumor cells. They induce anti-apoptotic effects as well as promote angiogenesis and metastasis [3]. There are many subfamilies of RTKs and only important ones as a therapeutic target are discussed here within the scope of the article.

### ErbBs family

ErbBs are typical RTKs recognized in avian erythroblastosis tumor virus and can encode for an abnormal form of the human EGFR [43]. Examples of this family include EGFR/ErbB-1, ErbB-2/HER-2/neu, ErbB-3/HER-3, and ErbB-4/HER-4 [43-45]. Activation of EGFR requires its binding with EGF peptide which in turn induces growth, proliferation, and differentiation of numerous types of cell. In malignancy, tumor microenvironment produces certain EGFR ligands which constantly stimulate EGFR. The second reason for such stimulation is the result of a mutation in EGFR itself that locks the receptor in a state of continual activation [45-47]. Constitutively, activated state of EGFR, HER-2, and HER-3 in epithelial tumors consequence in aggressive tumor behaviors like initiation and extension of tumor growth, metastasis and chemoresistance, finally results into poor patient outcome [45,48,49]. Non-small-cell lung cancer (NSCLC), oral squamous carcinoma, colorectal cancer, and breast cancer exhibits EGFR overexpression [43,44,50]. Overexpression of HER-2 protein occurs in approximately 25-30% of breast cancers and impend poor clinical outcome [49]. HER-2 and HER-4 receptors receive extracellular signals from their ligands *viz*. EGF, betacellulin, epiregulin, and transforming growth factor, as well as neuregulins which only bind to HER3 and HER4 [51]. HER3 receptor upregulation is usually seen in various malignancies such as mammary tumor, colorectal carcinoma, squamous cell carcinoma of the head and neck (SCCHN), uveal melanoma, and gastric, prostate, ovarian, and bladder cancers [51]. Thus, obviously, ErbBs receptors have emerged as a principal target for therapeutic intervention. The first EGFR-TK inhibitor approved by the US Food and Drug Administration (US FDA) for clinical use in advanced NSCLC was gefitinib in April 2003. Now it is licensed in more than 30 countries globally [38]. Erlotinib, a FDA approved first-line treatment for advanced NSCLC patients involving EGFR mutations, is also used as maintenance, second-line or third-line treatments following chemotherapy [52-54]. Lapatinib, when used as monotherapy, is found to be clinically useful in HER2-positive breast cancer. It is also active in the combination with trastuzumab as well as in trastuzumab-resistant patients. It is a dual tyrosine kinase inhibitor which selectively inhibits both EGFR/ErbB1 and HER2/ErbB2; whereas afatinib is a highly selective, irreversible inhibitor of EGFR, ErbB2/HER2, and ErbB4/HER4 [37]. Trastuzumab is a humanized immunoglobulin G1 (IgG1) monoclonal antibody targeting the Her2/neu receptor, recommended to treat the ErbB2 positive breast cancer, as monotherapy or in combination with other chemotherapy for adjuvant or palliative treatment [55]. It is also recommended as first-line treatment for ErbB2 positive gastric or gastro-esophageal junction carcinoma [56]. Cetuximab, a chimeric human-murine IgG1 antibody, is recommended to treat the SCCHN in combination with radiation therapy or other chemotherapy for the initial treatment of locally or regionally advanced [57-59] and colorectal cancer [60]. Panitumumab is full human IgG2 monoclonal and used to treat metastatic colorectal carcinoma that has progressed after treatment with other chemotherapy like oxaliplatin, irinotecan, and fluoropyrimidine [61].

## VEGFR

VEGFR family initiates signal cascades that stimulate angiogenesis, i.e., vascular development and lymphangiogenesis [62-64]. These receptors bind to VEGF ligands and initiate signaling transduction through dimerization and autophosphorylation [62,63]. Functions of three VEGFR family members *viz*. VEGFR-1 (Flt-1), VEGFR-2 (KDR/Flk-1), and VEGFR-3 (Flt-4) are known [62,64-66]. VEGFR-1 required for physiological angiogenesis at the early stage of embryogenesis, perhaps acting to trap and suppress VEGF [67]. The VEGFR-2 signaling plays an important role during development as well as neovascularization in both physiological and pathological conditions [62]. Upregulation of VEGFR-3 expression results in cardiovascular development during embryogenesis and lymphatic system development in adults [68]. The VEGF are reported to be of seven types, i.e., VEGF-A, VEGF-B, VEGF-C, VEGF-D, VEGF-E, VEGF-F, and placenta growth factor (PlGF). VEGF A, B and PlGF bind to VEGFR-1, VEGF A and E bind to VEGFR-2, and VEGF C and D bind to VEGFR-3 [62].

The tumors growth requires constant vascular growth with remodeling to solid tumors can grow beyond 1-2 mm^3^ in size [69,70]. Several VEGF-targeted agents administered either as single agents or in combination with chemotherapy have been shown to be beneficial for patients with malignancies in advanced-stage. The concept behind the development of such therapies was that they would inhibit new angiogenesis and thus tumors will lack necessary oxygen and nutrients [71]. After binding with VEGF ligands, activated VEGFR-2 results in increased vascular permeability along with a more migration and proliferation of endothelial cells, making it major target for therapy [72]. High VEGF expression results in highly disorganized and inefficient tumor vasculature. Thus, anti-VEGF agents can neutralize the effects of local VEGF and induce normalization of tumor vessels, resulting in a potential decrease in vascular volume and interstitial fluid pressure within the tumor allowing enhanced delivery of oxygen and cytotoxic therapies to the tumor [69].

Vatalanib, chemically an aminophthalazines class drug is a novel oral, small antiangiogenic molecule. It inhibits all three VEGFRs but more specifically VEGFR-1 and VEGFR-2. By hindering tyrosine kinase signaling, it blocks both angiogenesis and lymphangiogenesis [73]. It also reduces interstitial fluid pressure as monitored by dynamic MRI (magnetic resonance imaging) [74]. Vatalanib is under investigation for the treatment of patients with metastatic pancreatic cancer [75], colorectal cancer [76,77], NSCLC [78], metastatic breast cancer [79] and some other tumors. Sunitinib is another small-molecule, orally administered inhibitor of tyrosine kinases, and it is approved by the FDA for the treatment of renal cell carcinoma (RCC) [80,81] and imatinib-resistant gastrointestinal stromal tumor [82]. It acts on VEGFR as well as PDGFR [80].

Bevacizumab, a humanized monoclonal antibody, was the first US FDA approved therapy designed to inhibit angiogenesis [8,32,83]. It specifically targets and binds to VEGF-A protein, an isoform of VEGF that stimulates endothelial cell proliferation and subsequent migration, thereby inhibiting the process of angiogenesis [83]. It has been approved with or without other cancer therapy for the various clinical conditions such as metastatic colorectal cancer [84,85], non-squamous cell lung cancer [86], metastatic breast cancer [87], metastatic renal cell cancer [88], prostate cancer [66,89], and GBM [90].

## FGFRs

FGFs and FGFRs mediate a variety of cellular responses during embryonic development and in the adult organism. During embryonic development, FGFs play a critical role in morphogenesis by regulating cell proliferation, differentiation and cell migration, whereas in adults, they control development of the nervous system, regulates tissue repair, wound healing and tumor angiogenesis [91]. The FGFR family has five subfamily members: FGFR1, FGFR2, FGFR3, FGFR4, and FGFR5, out of which FGFR5 (FGFRL1) like others bind to FGFs but does not have tyrosine kinase domain [92]. Over-expression of FGFR or its activating mutations leads to constant and excess activation of the FGFR signaling pathway, resulting in increased proliferation and apoptosis evasion; thus performing the oncogenic functions [93]. Amplification or mutations of FGFR-1 have been reported in breast cancer (10%) [94], lung adenocarcinoma (21%) [95], oral squamous cell carcinoma (17.4%) [96], and GBM [97], whereas FGFR-2 amplification or mutations have been reported in about 2% breast cancer [98], 10% gastric cancer [99], 12% endometrial cancer [100], and lung cancer [98]. FGFR-3 mediated neoplasm has been reported in bladder cancer, multiple myeloma, cervical and prostate cancer [101-105], and FGFR-4 mutation has been reported in cases of bladder rhabdomyosarcoma and breast cancer [106-108].

PD173074 is a drug molecule with pyrido [2, 3-*d*] pyrimidine group as its core which interacts with ATP binding pocket of FGFR-1, and inhibits autophosphorylation of FGFR1. *In vivo* it blocks FGF2 induced angiogenesis [109] and it also blocks mitogenesis of tumor cells through G1-arrest, mediated by downregulation of cyclin D1 and cyclin D2 in breast cancer [110]. Drug molecule SU5402 is a narrow range tyrosine kinase inhibitor having indolin-2-one core and inhibits FGFR1, PDGFRB, and VEFGR2 tyrosine kinases [99,109]. SU5402 showed promising results with EGFR inhibitors in NSCLC [111], and alone in urothelial carcinoma [112]. Cediranib is a broad-range tyrosine kinase inhibitor and inhibits many receptors like FGFR1, PDGFRB, VEFGR2, PDGFRA, KIT, VEGFR-1, and VEGFR-3 tyrosine kinases [113]. Similarly, molecule Ki23057 is also a broad-range tyrosine kinase inhibitor, which suppresses FGFR1, FGFR2 and VEGF-2 tyrosine kinases [114]. In combination with other drugs, Ki23057 produced synergistic antitumor effects on scirrhous gastric carcinoma [114,115]. Other promising multikinase inhibitors are dovitinib, lucitanib, and ponatinib which are under investigation for endometrial cancer, FGFR1-amplified tumors and advanced squamous cell lung cancers, respectively [116].

FP-1039 (also known as GSK3052230 or HGS1036) is a receptor antagonist of FGFR1 [117,118]. A soluble fusion protein consisting of the extracellular domain of human FGFR1 fused to the Fc portion of human IgG1. Thus, FP-1039 prevents FGFR ligands, such as FGF1, FGF2 and FGF4 from binding to their cognate receptors, thereby inhibiting the activation of the related FGFR tyrosine kinases [118]. This molecule may also inhibit VEGF-induced angiogenesis. It targets the harmful FGFs that can cause cancer growth and also avoids inhibition of the hormonal FGFs and potential related toxicities [117,118]. FP-1039 mediated tumor inhibition occurs more specifically in FGFR1-amplified lung cancer and FGFR2-mutated endometrial cancer [117]. Neutralizing antibody-like PRO-001 are other therapeutic molecules which selectively inhibits the proliferation of FGFR3-transformed cells and induces apoptosis of FGFR3-expressing human myeloma cells. These anti-FGFR3 antibodies paved the way for the treatment of t(4;14) multiple myeloma [104]. There is considerable evidence underlining the relationship between FGFR4 mutation and carcinogens and studies has been attempted to focus possible correlation between FGFR4 (G388R) mutation and different cancers in the local population [119].

## IGFR

The insulin receptor (IR) and IGF1R belong to the same RTK family and both have ATP binding sites. Activation of the IGF pathway, by binding of the growth factors IGF-I and IGF-II to the receptors IGF-IR and IR, respectively, triggers complex signaling cascades that regulate cell growth, survival, proliferation, and differentiation in developmental stages [120]. Increased activation of IGF-I system is involved in many pathogenesis of various human cancers including breast, prostate, lung, and colon cancer [121]. This system is also known to play a crucial role in resistance to cancer therapies by preventing apoptosis, enhancing cell proliferation, and inducing expression of VEGF [122]. The immense expression of IGF-1R in cancer cells or tissues combined with its important role in cancer cell growth makes it a striking target to combat malignant diseases. Small molecule drugs that inhibit tyrosine kinase IGFR, i.e., BMS-754807, Insm-18 (NDGA), OSI-906 (linsitnib) and KW-2450 are under investigation in Phase I or II [123]. Some mABs, such as dalotuzumab (MK 0646), ganitumumab (AMG 479), and cixutumumab (A12), having same targets are under investigation in Phase III trial [123].

## HGFR

HGF is secreted by mesenchymal cells and stimulates epithelial cell proliferation, motility, scattering, morphogenesis and angiogenesis via phosphorylation of HGFR receptor, also known as c-Met or simply MET. HGF signals are essential for organ development in fetal life and failure of c-Met gene expression or HGF-neutralization leads to hypoplasia of many organs [124]. On the contrary, c-Met overexpression, with or without gene amplification has been reported in a variety of human cancers, including breast [125], lung [126], GI malignancies [127,128] and RCC [129]. Furthermore, high expression level of c-Met correlates with poor prognosis in many cancer cases, for example, breast, ovarian, cervical, gastric, head and neck, and NSCLCs [130]. Onartuzumab (OA5D5) and LY-2875358 are monoclonal antibody that bind with cMET receptors, whereas ficlatuzumab (AV-299) and rilotumumab (AMG-102) are monoclonal antibody that bind with HGF ligand; these molecules are under investigations to develop as targeted therapy. Similarly, tivantinib, cabozantinib (XL-184) and E-7050 are tyrosine kinases inhibitors being developed as small molecule drugs [130].

### RTK-like orphan receptor (ROR)

The ROR was named as orphan receptors because there endogenous ligands for their activation were unknown [131]. The human ROR family includes two members, ROR1 and ROR2, and regulates skeletal and neuronal development. Their gene is highly expressed during early embryonic development [132,133] but expressed at very low levels in adult tissues. The RTK ROR1 has been shown to be overexpressed in chronic lymphocytic leukemia [134,135]. Ovarian stem cells are known to express ROR1. Silencing of ROR1 suppress polycomb ring finger oncogene expression in human ovarian cancer. An anti-ROR1 monoclonal antibody molecule (UC-961) utilizes the same target [136]. Inhibition of the RTK ROR1 by anti-ROR1 mABs and siRNA induced apoptosis of melanoma cells also has been reported [137].

### Latest progress in cancer therapeutics

Trebananib (AMG 386), is a novel peptibody which neutralizes the interaction between angiopoietins (Ang1/2) and their Tie2 receptors and thus is a promising agent targeting signaling pathways of angiogenesis in ovarian cancer [138].

PDGF-mediated signaling mechanism, as a basis for cancer development, is also reported to be involved in regulation of growth and survival of different cell types. Dysregulation of spatiotemporally controlled PDGF-induced signaling has been adequately reviewed elsewhere and are a potential target for future cancer therapeutics [139,140].

## Conclusions

RTKs are important mediators of the signaling cascade, determining key roles in diverse biological processes such as growth, differentiation, metabolism, and apoptosis in response to external and internal stimuli. Enhanced signal generation, oncogenic mutations of RTKs, gene amplifications of RTKs and oncogene fusions involving RTKs may lead to oncogenic activation of RTKs results in cancer. RTKs targeted therapy block specific pathways related to carcinogenesis and tumor growth by inducing apoptosis of cancer cells, blocking specific enzymes and growth factor receptors involved in cancer cell proliferation, or modifying the function of proteins that regulate gene expression and other cellular functions, rather than by simply interfering with all rapidly growing cells. To target the RTK mainly two forms are used: Small-molecule drugs and mABs. Recent advancement in knowledge of activation of RTKs, their signaling pathways and antagonists provides key breakthrough in development of novel anticancer drugs. Still, there is gap in knowledge about functioning of the RTK subfamilies which needs to be fulfilled in coming future for implications of personalized medicine by targeting a specific cellular pathway within the tumor.

## Authors’ Contributions

All authors contributed the relevant literature in preparation of this review. All authors read and approved the final manuscript.
